# Significant rise of the prevalence and clinical features of childhood asthma in Qingdao China: cluster sampling investigation of 10,082 children

**DOI:** 10.1186/1471-2458-14-1002

**Published:** 2014-09-26

**Authors:** Rongjun Lin, Renzheng Guan, Xiaomei Liu, Baochun Zhao, Jie Guan, Ling Lu

**Affiliations:** The Affiliated Hospital of Qingdao University, Qingdao, 266003 China; Qingdao Hiser Medical Group, Qingdao, 266033 China

## Abstract

**Background:**

Recent investigations suggested that the trend of childhood asthma has been stabilizing or even reversing in some countries. The observation provides contrast to our experience. Thus, the study aimed to investigate the prevalence and clinical features of asthma in children aged 0–14 years in Qingdao China, determine the changes of childhood asthma in China, and discover evidence that can allow better diagnosis and treatment of childhood asthma.

**Methods:**

A cluster sampling method was used. We randomly extracted the investigation clusters from schools, kindergartens, and communities in Qingdao. Subsequently, we interviewed the members of the clusters using a questionnaire from the International Study of Asthma and Allergies in Childhood (ISAAC) to find children with asthmatic symptoms. After determination by the doctors, more details on the asthmatic children were obtained by asking questions from the National Epidemiology Study of Asthma and Allergies in China questionnaire to obtain more details. We intended to survey 10,800 children. However, the actual number of children was 10,082.

**Results:**

The prevalence of asthma in Qingdao children aged 0–14 years was 3.69%. The prevalence among male children was higher than in female (*χ*2 = 24.53,P < 0.01). Among the asthmatic children, 68.0% had their first attack when they were less than three years old. Moreover, 71.2% once suffered respiratory tract infections. For 95.7% of asthmatic children, the asthma attack was first manifested as cough. Asthmatic children who used inhaled corticosteroids (ICS) only accounted for 46%.

**Conclusions:**

The prevalence of asthma in children aged 0–14 years in Qingdao China increased significantly based on data obtained ten years ago (2000). Respiratory tract infections were the most important precursors of asthma attack. The attack was most commonly manifested as cough. The treatment, especially the use of ICS, was more rational. However, a certain difference was found, which has yet to be contrasted with the Global Initiative for Asthma (GINA) project.

## Background

Asthma in children has become an increasingly remarkable public health problem worldwide. In 1998, an ISAAC article reported that more than 20% of children once suffered from wheezing in Australia, New Zealand, Oman, Peru, Singapore, and the UK [1]. Additionally, 9.1% of US children (6.7 million) had asthma in 2007 [2]. As early as 2002, WHO defined asthma as the most common chronic disease in children according to large-scale investigations and analysis [3]. The prevalence of childhood asthma in China increased considerably in 1990s, which ranged from 0.93% in 1990 to 1.54% in 2000 [4]. In 2000, the prevalence of asthma in Qingdao was 2.67% [5]. No new investigation has been conducted in the past 10 years. To obtain the newest trends of childhood asthma in Qingdao China, a questionnaire-based survey was performed among 10,082 children, aged 0–14 years, as respondents.

## Methods

### Subject

Our study was conducted in Qingdao, a beautiful coastal city in eastern China with a temperate monsoon climate. Cluster sampling survey method was used. The required sample size, calculated with prevalence of 1.54% in 2000, included approximately 10,000 subjects. We randomly selected nine schools, seven kindergartens, and three communities in Qingdao to be investigated using the lottery method. Our subjects were the children born between July 1, 1996 and June 30, 2010, which had a total of 10,800 children.

### Methods and quality-control measures

Investigators were doctors or medical students, who were trained before the program. ISAAC and National Epidemiology Study of Asthma and Allergies in China questionnaires were applied in the survey. Two rounds of surveys were launched. First, all the respondents completed the screening questionnaire (ISAAC questionnaire), in which patients suspected of asthma were selected. Second, after the diagnosis by pediatricians in our hospital, asthma patients were asked to complete a second in-depth questionnaire for asthma. A control group that matched the patients was formed to complete a second in-depth questionnaire for non-asthma (National Epidemiology Study of Asthma and Allergies in China questionnaire). In the case of young children, the questionnaires were completed by students or their parents. All the papers were checked by investigators before adoption. The survey started in September 2010 and finished in March 2011.

The following diagnostic criteria of asthma were used: (1) respondents had been diagnosed with asthma by professional pediatricians of three A-grade hospitals and received asthma treatment, or (2) respondents were diagnosed in our hospital using the diagnostic criteria of GINA 2012 [6].

The investigation was performed in accordance with the Declaration of Helsinki and approved by the Medical Ethics Committee of the Affiliated Hospital of Qingdao University. Each questionnaire was approved by the child’s guardian.

### Data management and analysis

Data were double entered using epi-info system. Statistical analyses were performed using the SPSS 17.0 software. *χ*2 test was applied for data comparison between groups. P values < 0.05 were regarded as statistically significant in all analyses.

## Results

### Prevalence of asthma in Qingdao

The prevalence of childhood asthma in Qingdao was 3.69%. Overall, 10,082 children were investigated. Among these children, 373 were diagnosed with asthma. From this group of children, 57 were identified to have cough variant asthma, which accounted for 15.3%. The prevalence in children aged 3–7 was higher than the average. Table 1 shows the prevalence of asthma in different ages.

**Table 1:** **The prevalences of asthma in different ages**

Age (Year)	Number	Asthma children	Prevalence (%)
~1	243	2	0.82
~2	351	12	3.42
~3	344	26	7.56
~4	595	100	16.81
~5	638	61	9.56
~6	650	63	9.69
~7	1005	43	4.28
~8	701	16	2.28
~9	1076	14	1.30
~10	705	5	0.71
~11	924	15	1.62
~12	774	7	0.90
~13	915	2	0.22
~14	947	5	0.53
~15	214	1	0.47
Total	10082	372	3.69

### Gender and age difference

Among the 10,082 respondents, 5,126 were males and 4,956 were females. The ratio of male to female was 1.03:1. However, the prevalence of asthma in males was 4.60%, and in females was 2.74%, which shows a clear difference (*χ*2 = 24.53,P < 0.01).

### Age of the first attack and initially diagnosed as asthma

Based on first asthmatic attack, the children were divided into three age groups: <3 y, 4–6 y, and 7–14 y. The incidents of first attack were 79.3%, 17.7%, and 3%, respectively. Based on the same grouping, 39.2%, 56.5%, and 4.3% of the children of each group were initially diagnosed as asthmatic.

### Precursors of asthma attack

Asthma attacks in 71.2% of the asthmatic children were caused by respiratory tract infection. Other precursors included cold air, exercise, eating fish, and smoke, which accounted for 42.2%, 28.2%, 13.7%, and 9.4%, respectively.

### Signs of attack and clinical manifestations

Runny nose, sneezing, and nasal congestion were identified as the most common signs of asthma attack (63.4%, 62.9%, and 53.0%, respectively). Table 2 presents the details. In all clinical manifestations, cough was observed on 95.7% of the patients and 35.8% of the asthmatic children had cough for more than one month. Other manifestations were wheezing (82.3%) and suffocation (41.4%). Table 3 shows every clinical manifestation and its incidence. Most asthmatic children (72.3%) felt that the asthma attack was tolerable, but 17.5% had to visit the emergency room because of severe asthma attacks.

**Table 2:** **Incidence of signs of attack in 372 asthma children**

Signs	Number	Incidence(%)
Runny nose	236	63.4
Sneezing	234	62.9
Nasal congestion	197	53.0
Itchy noes	145	39.0
Itchy eyes	99	26.6
Itchy throat	47	12.6
Others	37	9.9

**Table 3:** **Incidence of manifestations in 372 asthma children**

Manifestations	Number	Incidence(%)
Cough	356	95.7
Wheezing	306	82.3
Suffocation	154	41.4
Night waking	142	38.2
Chest tightness	69	18.5
Prolonged expiratory	68	18.3
Dyspnea	13	3.5
Sweat	5	1.3
Orthopnea	4	1.1
Dysphagia	2	0.5
Cyanosis	1	0.3

### Treatment and economic cost

**Figure 1:**
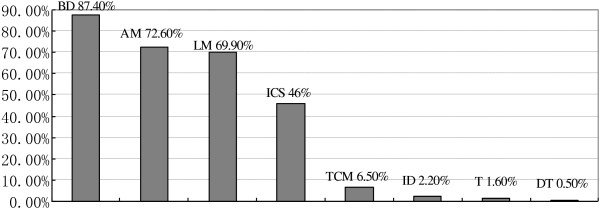
**Drugs used in asthmatic children.**

Figure 1 shows the high use of quick-relief medications, such as bronchodilators (87.4%), and low use of preventative medication, such as ICS (46%). Among the patients, 72.6% and 69.9% used anti-allergy medicine and leukotriene modifiers, respectively. Traditional Chinese medicine was used by 6.5% of the patients. Immunomodulators, theophylline, and desensitization therapy were rarely used. In an acute attack, 29.8% patients took antibiotics and 10.5% were given systemic application of steroid.

This disease entailed significant costs for a number of asthmatic children and their families; 71.8% of the parents spent ¥2000–5000 (approximately $ 300–750) annually for their asthmatic children; 14.2% spent less than 2000, and 14% spent more than ¥5000. Nevertheless, almost all parents (99.7%) expressed their capability to afford the treatments.

## Discussion

### Possible reasons for high prevalence in Qingdao

After 2000, results of several studies on the prevalence of childhood asthma showed no changes [7–9] or even significant decreases [10, 11]. By contrast, more studies indicated an increase in the prevalence [12–16]. Our study agrees with the latter. The prevalence of childhood asthma in Qingdao (3.69%) was considerably higher compared with the data gathered in 2000 (2.67%) [5], The trend that was also observed in other Chinese cities [17]. The prevalence of childhood asthma in Qingdao was higher than the national average (3.02%) [18].

Environmental, climate, and industrial factors may be related to the high prevalence of childhood asthma in Qingdao. First, the moist air in Qingdao, a beach city, provides a good environment for dust mites and mildew, which may be the most important allergens of asthma [19]. Second, Qingdao residents are partial to seafoods, which easily cause allergic disease. Third, the farming environment, which offered protective effect on the development of asthma in children [20], has been affected by the rapid industrialization, which also increased air pollution. The environmental and climate factors definitely contribute to the causes of asthma [21, 22]. LIU Yan-li reported that mites were the major allergens, and eating seafoods was an important risk factor for asthmatic children in Qingdao [23]. Additionally, many patients informed us that they felt much better in another city. However, the aforementioned potential reasons require further study. We are working hard to complete such study.

### Improvement in diagnosis of asthma

In recent years, an increasing number of Chinese doctors have become familiar with GINA, which resulted in significant improvement in the diagnosis of asthma. In the survey, 97% of asthmatic children had their first attack at <6 y. Meanwhile, 95.7% were initially diagnosed with Asthma at <6 y. Most (98.7%) asthmatic children can be inferred to have received accurate diagnosis at the initial period of the disease.

### Role of respiratory tract infection in asthma

Among the asthmatic children, 71.2% had suffered asthma attack after contracting respiratory tract infection. Clearly, the infection of the respiratory tract had a close relationship with asthma. The possible mechanism was that the pathogen injury of the airway epithelial, which caused the airway to become hyper responsive and induced asthma to occur more easily. Dr. Gern reported that respiratory syncytial virus and rhinovirus were the main viruses causing asthma attacks in infants and older children, respectively [24]. Atypical microorganisms were also reported to influence the occurrence of an asthma attack [25]. Therefore, asthmatic children should be protected from respiratory infection to reduce the risk of asthma attack.

### Problems in treatment

Epidemiological data indicated that asthma control was suboptimal in both developed and developing countries [26–28]. The same problem was also found in our survey. We found that only 46% of asthmatic children in Qingdao China used ICS, which was the main and basic control medicine for asthma [6]. However, this percentage denotes considerable progress compared with data from 2000, in which only 19.6% of asthmatic children used ICS [5]. Compared with the use of bronchodilators (87.4%), anti-allergy (72.6%), and leukotriene modifiers (69.9%), the resistance to the use of ICS was stronger, which may have come from parents. The possibility of steroid-related side effects could be the first reason. Numerous parents refused immediately when medicine with steroid was discussed. Other parents put forward the concern on whether ICS would result in a short or unhealthy child. Lack of apparent efficacy may be the second reason. Many parents complained over the uselessness of ICS as proven by the lack of immediate change in the condition of their children. Moreover, bronchodilators quickly transformed the frowns on children’s faces to smiles. Based on following observations, we believe economic factors did not play a critical role in the parents’ medicine choice. As mentioned earlier, almost all parents expressed their capability to afford the treatments. Moreover, the daily cost of leukotriene modifiers was not lower than that of ICS in China. GINA has proven that ICS is the best medicine for asthma control. A series of research demonstrated that ICS produced minimal side effects [6]. Many parents were worried over the height of their children. In early 2000, the New England Journal of Medicine revealed that inhaling medium or low dose ICS did not affect the height of asthmatic children [29]. Nevertheless, guiding parents and their children toward acceptance of this idea remains a difficult task to accomplish.

## Conclusions

Our survey showed that the present prevalence of asthma in Qingdao is significantly higher compared with its occurrence ten years ago. This observation is consistent with the general trend in China. Thus, childhood asthma, which remains an important public health concern, demands more attention today and in the future. Environmental and industrial changes may have contributed to this trend. Less respiratory tract infection may mean less asthma attack. Despite the increasing reliance on ICS, the fulfillment of this goal seems to have become a more challenging process. Further studies are needed to discern the possible reasons that could fully explain the observation.

## Author’s information

Renzheng Guan is co-first author.
